# 晚期非小细胞肺癌免疫治疗的一线联合治疗

**DOI:** 10.3779/j.issn.1009-3419.2018.12.11

**Published:** 2018-12-20

**Authors:** 晓潇 彭, 清 周

**Affiliations:** 510080 广州，广东省人民医院（广东省医学科学院），广东省肺癌研究所 Guangdong General Hospital (Guangdong Academy of Medical Sciences), Guangdong Lung Cancer Institute, Guangzhou 510080, China

**Keywords:** 程序性死亡分子1/程序性死亡配体1抑制剂, 肺肿瘤, 一线联合, 免疫肿瘤治疗, Programmed death-1/programmed death-ligand-1 inhibitors, Lung neoplasms, First-line combination, Immuno-oncology

## Abstract

程序性细胞死亡分子1/配体1（programmed death 1/programmed death ligand 1, PD-1/PD-L1）抑制剂已成为晚期非小细胞肺癌（non-small cell lung cancer, NSCLC）重要治疗手段之一。目前已有一部分NSCLC患者可以接受PD-1抑制剂单药作为一线免疫肿瘤治疗（immuno-oncology，IO，又简称免疫治疗），但应用时的诸多限制使可成为IO一线单药治疗候选者的人群有限。使IO一线治疗可让更广人群的更多获益，多项研究正聚焦于IO与其他药物在NSCLC中的联合应用。本文回顾了近年来IO一线联合治疗的最新临床数据，提示在PD-1/PD-L1抑制剂的基础上，联合其他IO、化疗、抗血管生成药物、靶向治疗或放疗可能产生抗肿瘤协同效应，有望使更多初治患者获益。

## Abstract (en)

Programmed death 1/programmed death ligand 1 (PD-1/PD-L1) inhibitor has become one of the important treatment options for patients with advanced non-small cell lung cancer (NSCLC). However, only a small subset of patients with NSCLC can currently receive single-agent PD-1 inhibitors as first-line therapy, for the limitations of population selection exclude most patients from immuno-oncology (IO) monotherapy. In order to expand the candidate population for IO first-line treatment and make more newly diagnosed patients benefit from IO treatment, a series of studies are focusing on the combination of IO and other drugs in NSCLC. We reviewed the latest clinical data of IO first-line combination therapy in recent years, suggesting that on the basis of PD-1/PD-L1 inhibitors, combined with other IO, chemotherapy, anti-angiogenic drugs, targeted therapy or radiotherapy may produce synergistic anti-tumor effects. It is expected to benefit more newly diagnosed patients.

以程序性死亡分子1（programmed death 1, PD-1）抑制剂为代表的免疫肿瘤治疗（immuno-oncology，IO，又简称免疫治疗）在非小细胞肺癌（non-small cell lung cancer, NSCLC）中的一线研究数据令人瞩目，并已经成为无驱动基因、程序性死亡配体1（programmed death ligand 1, PD-L1）高表达（≥50%）、不可手术的局部或转移性晚期NSCLC患者新的标准一线治疗手段之一，但应用时的诸多限制使可成为IO一线单药治疗候选者的人群有限。为使IO一线治疗可让更广人群更多获益，多项研究正聚焦于IO与其他药物在NSCLC中的联合应用，并取得了令人鼓舞的进展。本文回顾了近年来IO联合治疗的最新进展与探索方向，旨在为相关领域的临床医生提供一定参考。

## 1 IO在NSCLC中的抗肿瘤作用机制

目前仅PD-1/PD-L1免疫检查点抑制剂获批用于治疗NSCLC。PD-1/PD-L1通路是NSCLC肿瘤细胞逃避免疫监测的重要方式之一。PD-1广泛表达于肿瘤浸润性淋巴细胞（tumor-infiltrating lymphocyte, TIL）、B细胞、自然杀伤细胞、单核细胞和树突状细胞（dendritic cell, DC）表面^[1, 2]^，通过与其配体PD-L1的结合，抑制肿瘤微环境中T细胞的增殖、活性和存活，减少干扰素-γ（interferon γ, IFN-γ）、肿瘤坏死因子-α（tumor necrosis factor α, TNF-α）、白介素-2等免疫效应分子的表达，从而使T细胞功能失调并加速耗竭，减弱抗肿瘤效应^[3]^。PD-L1可表达于多种肿瘤细胞表面，如肺癌、胃癌、结直肠癌、肾癌、膀胱癌等^[4]^。这些肿瘤细胞可通过多个原癌基因信号通路激活PD-L1的表达，或通过浸润免疫细胞持续表达的炎性细胞因子（如IFN-γ等）造成PD-L1的适应性上调^[4]^。因此，PD-1/PD-L1通路在降低肿瘤特异性T细胞活性、抑制免疫系统的抗肿瘤效应等方面发挥重要作用，抑制这一通路可恢复T细胞对肿瘤的杀伤活性、控制肿瘤生长。

## 2 IO单药治疗在NSCLC中的一线应用现状与不足

IO治疗的优势在于一旦患者产生应答，其应答效应往往持久，甚至数年之后仍可有效，且获得性耐药发生较低（例如，CA209-003研究最新随访数据显示，75%存活的晚期NSCLC患者接受PD-1抑制剂nivolumab治疗后5年时仍有应答，患者5年生存率可达16%）^[5]^。但另一方面，IO治疗原发性无应答患者比例较高（二线单药治疗不考虑PD-L1水平时，总体人群有效率为10%-20%）^[6]^。目前仅PD-1抑制剂pembrolizumab获批成为PD-L1≥50%、驱动基因阴性晚期NSCLC患者的一线治疗。这一IO单药治疗无疑可使部分PD-L1高表达的NSCLC患者获益，但也存在诸多限制。

首先，尽管PD-L1高表达人群接受pembrolizumab一线单药治疗时有效率可达45.5%且生存获益显著，但这部分患者仅占所有NSCLC初治人群的30%^[7, 8]^。根据美国国家综合癌症网络等权威指南的建议，排除PD-L1低表达（仅约30%的NSCLC患者≥50%）、表皮生长因子受体（epidermal growth factor receptor，EGFR，约50%的亚洲患者）与间变性淋巴瘤激酶（anaplastic lymphoma kinase，ALK，约5%的NSCLC患者）阳性突变以及体能状态（performance status, PS）评分较差的患者之后，最终仅约10%的患者可能成为IO一线单药治疗的适宜人群^[8]^；其次，pembrolizumab给予治疗之前，需对患者PD-L1表达状态进行伴随诊断，测得患者PD-L1表达≥50%（22C3试剂盒检测）方可用药，其中的报告回复时间在真实环境中是否会影响患者生存转归有待证实^[8]^；再次，即使是PD-L1≥50%的患者，亦有半数以上对PD-1抑制剂无效，提示这类患者可能存在其他免疫抑制机制，如细胞毒性T细胞抗原-4（cytotoxic T-lymphocyte-associated antigen 4, CTLA-4）、吲哚胺2, 3-双加氧酶（indoleamine 2, 3-dioxygenase, IDO）过表达等^[9]^。临床前研究^[9]^证实，对不同的、互为补充的免疫抑制通路的双重或多重联合拮抗，可能产生协同作用，使更多单药治疗“无效”患者产生抗肿瘤效应。因此，为进一步提高疗效并使更多患者获益，针对PD-1/PD-L1抑制剂最优生物标志物的探索与联合治疗新方向的探索开始成为研究者们关注的新焦点，有关IO与多种治疗手段一线联合应用的研究不断涌现。

## 3 IO联合方案在NSCLC中的一线应用现状

### 3.1 IO+IO

PD-1与CTLA-4同属免疫检查点分子，但作用机制不同，并在免疫反应的不同阶段对T细胞的活化发挥负向调控作用。CTLA-4可在初始T细胞的最初活化阶段阻止T细胞的激活与效应功能，而PD-1则在免疫反应的较晚期作用于已活化的T细胞，抑制T细胞的活化程度与细胞毒性^[3]^。因此，CTLA-4与PD-1的双重拮抗可起到协同作用，在免疫反应早期诱导大量T细胞的活化与增殖、晚期恢复耗竭T细胞的免疫活性，并降低调节T细胞（regulatory T cell, Treg）介导的免疫抑制效应^[3]^。动物模型研究显示，PD-1/CTLA-4联合拮抗可增强免疫系统抗肿瘤效应、优化效应T细胞/Treg比例、增加IFN-γ/TNF-α双重分泌性CD8^+^ T细胞出现频率，将肿瘤微环境由免疫抑制转变为TIL浸润与免疫清除状态^[10]^。基于此，有关PD-1/CTLA-4联合治疗的大型临床研究陆续开展。

CheckMate 012研究中有关nivolumab+ipilimumab联合队列的最新结果令人瞩目^[11]^。共77例未经化疗、PS评分0分-1分的Ⅲb期/Ⅳ期NSCLC患者随机接受nivolumab 3 mg/kg *Q2W*+ipilimumab 1 mg/kg *Q12W*（N+I *Q12W*, *n*=38）或*Q6W*（N+I *Q6W*, *n*=39）治疗，最新的2年总生存（overall survival, OS）分析显示^[12]^，N+I Q12W与Q6W两组患者的汇总客观缓解率（objective response rate, ORR）达到43%，中位无进展生存期（progression-free survival, PFS）为8.0个月，1年OS率为76%。根据PD-L1 < 1%、≥1%和≥50%进行分层分析后发现，PD-L1高表达人群的生存获益更为显著（具体有效性数据见表 1）。研究结果提示，nivolumab+ipilimumab一线联合治疗晚期NSCLC患者安全性可控，有效性显著且持久。

**1:** CheckMate 012研究N+I队列的有效性结果更新^[12]^ Updated efficacy results of cohort N+I from CheckMate 012^[12]^

Cohorts	ORR (%)	Median DOR^a^ (95%CI) (mo)	Median PFSa (95%CI) (mo)	1-year OS rate (%)	2-year OS rate (%)^[13]^
N+I Q12W^b^ (*n*=38)	47	NR (11.3, NA)	8.1 (5.6, 16.7)）	83	56
N+I Q6W^c^ (*n*=39)	38	NR (11.2, NA)	3.9 (2.6, 13.4)	69	42
Pooled (*n*=77)	43	NR (14.0, NA)	8.0 (4.1, 13.2)	76	49
PD-L1≥50% (*n*=13)	92	NR (4.2, NA)	NR (7.8, NA)	100	62
PD-L1≥1% (*n*=46)	57	NR (11.2, NA)	12.7 (7.8, 23.0)	87	58
PD-L1 < 1% (*n*=19)	21	NR (NA)	3.3 (2.3, 5.3)	53	--
^a^Using the *Kaplan-Meier* method; ^b^Median follow-up was 15.8 months; ^c^Median follow-up was 16.1 months. NA: not available; NR: not reached; --: not reported; ORR: objective response rate; PFS: progression-free survival; OS: overall survival; DOR: duration of response.					

Checkmate-227 Ⅲ期研究（第1部分）^[14]^进一步在高肿瘤突变负荷（≥10突变/Mb）的Ⅳ期或复发性NSCLC患者中探索了nivolumab+ipilimumab一线治疗对患者生存预后的影响。研究显示，在高肿瘤突变负荷、未经化疗的晚期NSCLC患者中，nivolumab+ipilimumab一线治疗（*n*=139）较化疗（*n*=160）可使患者1年PFS率（42.6% *vs* 13.2%）和中位PFS期（7.2个月*vs* 5.5个月，HR=0.58；*P* < 0.001）显著改善，ORR也有所提高（45.3% *vs* 26.9%）。Nivolumab+ipilimumab相较于化疗的生存获益在各亚组中广泛一致，包括不同PD-L1水平（≥1%或 < 1%）、不同组织学分型（鳞癌或非鳞癌）和不同体力状态评分（0分或≥1分）的人群。Nivolumab +ipilimumab组和化疗组的3级-4级治疗相关不良事件（treatment related adverse event, TRAE）发生率分别为31.2%和36.1%。研究进一步证实了nivolumab+ipilimumab联合治疗在NSCLC中的应用前景，并提示突变负荷可能成为适宜人群筛选的标志物之一。

另一项有关PD-L1抑制剂durvalumab与CTLA-4抑制剂tremelimumab联合用药的Ib期研究纳入了102例未经免疫治疗的局部晚期或转移性NSCLC患者（其中6%未经系统化疗），给予不同剂量的联合治疗^[15]^。结果显示，28%的患者因TRAE终止了治疗，剩余63例疗效可评估患者的ORR为17%。有关durvalumab与tremelimumab的一线联合治疗Ⅲ期研究MYSTIC（NCT02453282）、NEPTUNE（NCT02542293）等仍在进行，目前尚无结果发表。

### 3.2 IO+化疗

既往研究显示，传统的化疗药物可能通过不同途径激活抗肿瘤免疫反应，包括诱导具免疫原性的肿瘤细胞死亡，或扰乱肿瘤细胞逃避免疫监控的策略^[16]^。例如，蒽环类药物治疗的肿瘤细胞可分别通过嘌呤受体或Toll样受体-4激活TIL和DC，从而使其成为“免疫性肿瘤细胞”，激活抗肿瘤免疫反应^[17]^；吉西他滨也可通过诱导肿瘤细胞凋亡、增强CD8^+^ T细胞的交叉提呈、修复肿瘤浸润性DC的抗原提呈缺陷等多效性手段激活免疫效应^[16]^。因此，IO与化疗的联合用药可能具有协同作用，但与化疗药物的类型、剂量和用药方案（同时或序贯）密切相关^[16]^。

KETNOTE-021 G队列Ⅱ期研究的最新数据显示^[18]^，对于晚期非鳞癌NSCLC患者，在标准一线化疗（卡铂+培美曲塞，PC）基础上联合pembrolizumab可显著改善非鳞癌患者的ORR并延长其PFS。中位随访2年时，pembrolizumab+PC（*n*=60）组的ORR、PFS和OS均显著优于PC化疗组（*n*=63），其中ORR分别为57%和30%（*P*=0.001, 6），中位PFS分别为24.0个月与9.3个月（HR=0.534, *P*=0.004, 9），中位OS则分别为未到达与21.1个月（HR=0.56, *P*=0.015, 1）。联合组和化疗组3级-5级不良事件（adverse event, AE）发生率分别为41%与27%。根据KEYNOTE 021研究的前期结果，食品药品监督管理局（Food and Drug Administration, FDA）已于2017年5月批准pembrolizumab联合化疗用于非鳞癌NSCLC的一线治疗。但值得注意的是，对于PD-L1表达在1%-49%范围内的患者，pembrolizumab联合化疗组的ORR反而低于单用化疗组（26% *vs* 39%）^[19]^，这一结果与PD-L1 < 1%、PD-L1≥50%的人群截然相反，是样本量过小造成的偶然偏倚，又或是这部分低表达的患者更适合接受化疗或其他联合治疗？后续的大样本随机对照研究或可回答这一问题。

Ⅲ期研究KEYNOTE 189和KEYNOTE 407分别探索了pembrolizumab联合化疗一线治疗在非鳞癌和鳞癌NSCLC人群中的有效性与安全性。KEYNOTE 189研究中位随访10.5个月时^[20]^，pembrolizumab+含铂化疗（*n*=410）组的ORR、PFS和12个月OS率均显著优于安慰剂+含铂化疗组（*n*=206），其中ORR分别为47.6%和18.9%（*P* < 0.001），中位PFS分别为8.8个月与4.9个月（HR=0.52, *P* < 0.001），12月OS率估计值则分别为69.2%与49.4%（HR=0.49, *P* < 0.001）。在PD-L1表达 < 1%、1%-49%和≥50%的非鳞癌人群中，pembrolizumab+化疗组12个月OS率的HR值较安慰剂+化疗组分别为0.59、0.55和0.42，显示无论PD-L1表达水平如何，非鳞癌NSCLC患者均可从pembrolizumab联合治疗中得到总生存获益。Pembrolizumab+化疗组和安慰剂+化疗组≥3级AE的发生率分别为67.2%与65.8%。KEYNOTE 407研究的最新中期分析则显示^[21]^，中位随访7.7个月时，pembrolizumab+化疗（*n*=101）较安慰剂+化疗（*n*=103）显著提高转移性鳞癌NSCLC患者的ORR（58.4% *vs* 35.0%, *P*=0.000, 4）。Pembrolizumab+化疗组与安慰剂+化疗组持续缓解≥6个月的患者比例分别为65.8%与45.6%，≥3级AE的发生率则分别为64.4%与74.5%，且无新型AE发生。Pembrolizumab+化疗一线联合方案在鳞癌NSCLC人群中的初步有效性和耐受性良好。

CheckMate 012研究^[22, 23]^中nivolumab+含铂双药化疗的结果显示，nivolumab（5 mg/kg或10 mg/kg）联合含铂双药方案治疗NSCLC患者的ORR可达46%，疾病控制率达89%，中位OS可达19.2个月，1年、2年、3年OS率分别为71%、37%和25%。所有联合组选定3级-4级AE（如内分泌、胃肠道、肝、肾、肺毒性等）的发生率均≤7%。后续的CheckMate 227 Ⅲ期研究（第2部分）^[24]^将纳入更多未经系统抗癌治疗的Ⅳ期或复发性NSCLC患者，预计约480例患者随机接受含铂双药化疗或nivolumab+化疗联合方案。值得注意的是，这项研究目前也已经在中国开展。

另一项多中心、多臂Ib期研究（GP28328）^[25]^评估了PD-L1抑制剂atezolizumab联合一线化疗方案在多种肿瘤中的安全性和有效性。在NSCLC试验组中，共76例可评估的、未经化疗的局部晚期或转移性NSCLC患者随机接受了15 mg/kg atezolizumab *Q3W*联合下列方案之一治疗：卡铂+紫杉醇（TC）*Q3W*（*n*=25）、PC *Q3W*（*n*=25）和卡铂+白蛋白结合型紫杉醇（Nab-C）*QW*（*n*=26）。各组患者确定的ORR分别为36%、64%和46%；中位PFS分别为7.1个月、8.4个月和5.7个月；中位OS分别为12.9个月、19.3个月和14.8个月。该研究显示atezolizumab联合一线化疗方案可耐受，根据ORR、OS、PFS评估的临床有效性良好，提示atezolizumab与化疗之间或有潜在的协同作用。但样本量有限，需要进一步研究支持。后续Ⅲ期研究IMpower 130（atezolizumab+Nab-C, *n*=715）、IMpower 131（atezolizumab+TC/Nab-C, *n*=1, 025）和IMpower 132（atezolizumab+PC, *n*=568）均在进行之中。其中IMpower 131研究的最新结果^[26]^显示，atezolizumab+Nab-C较单用Nab-C可显著延长患者中位PFS（6.3个月*vs* 5.6个月；HR=0.715；*P*=0.000, 1），PD-L1表达阳性的患者PFS获益更为显著。初步OS结果尚未发表。Atezolizumab+TC/Nab-C治疗患者的安全性特征与既往报道一致，无新型AE发生，TRAE发生率与Nab-C组也无显著差异。

联合用药的时机与不良反应的处理，将是IO与标准化疗联合用药时需考虑的问题。最佳联合时机应最小化两者的相互拮抗作用并最大化协同效应，目前一个可能的策略是在手术和化疗有效缩小肿瘤体积至微小残留病灶存在时给予IO治疗，从而使过大的肿瘤体积对抗肿瘤免疫效用的负面影响降至最低，同时又使化疗可调节残余肿瘤细胞的免疫表型^[16]^。如何寻找免疫治疗与化疗联合最适宜的用药人群、如何更好地平衡协同抗肿瘤效应与不良反应，也将成为这一领域未来的挑战^[16]^。

### 3.3 IO+抗血管生成药物

血管内皮生长因子（vascular endothelial growth factor, VEGF）通路不仅可以在肿瘤血管化过程中发挥作用，也是肿瘤细胞发生免疫逃避的机制之一^[27]^。既往研究表明，抗肿瘤免疫反应与肿瘤血管生成的相关通路存在相互影响。VEGF可促进免疫抑制性细胞群体的产生（如Treg和髓细胞源性抑制细胞等）、限制DC等抗原提呈细胞的成熟和效应T细胞的功能、阻碍肿瘤特异性T细胞和其他免疫效应细胞向肿瘤微环境的浸润和迁移，因此，VEGF抑制剂可通过拮抗上述多种途径增强机体的抗肿瘤免疫效应。另一方面，免疫检查点抑制剂也可介导肿瘤血管病变，进而导致肿瘤坏死和单核细胞大量浸润至肿瘤内部^[27]^。临床前研究与其他肿瘤中的临床研究均证实，IO与VEGF抑制剂（如贝伐珠单抗）可能产生协同作用^[27]^。

IMpower 150研究^[28]^中位随访15个月的最新结果显示，在无*EGFR*/*ALK*突变的意向治疗人群中，atezolizumab +TC+贝伐珠单抗组患者（*n*=356）经研究者评估的中位PFS和中位OS均显著长于贝伐珠单抗+TC组（*n*=336；中位PFS：8.3个月*vs* 6.8个月，*P* < 0.000, 1；中位OS：19.2个月*vs* 14.4个月，*P*=0.026, 2），1年PFS率亦较高（37% *vs* 18%）。比较有意思的是，亚组分析显示无论肝转移及*EGFR*、*ALK*或*KRAS*突变状态如何，atezolizumab+TC+贝伐珠单抗均可使患者获益^[28, 29]^。两组患者严重TRAE发生率分别为25.4%和19.3%，联合方案的AE特征与既往单一药物的已知安全性一致。

有关PD-1/PD-L1抑制剂与VEGF抑制剂联合用药的研究还包括CheckMate 012（nivolumab+TC+贝伐珠单抗维持治疗）等，亦在进行之中。

### 3.4 IO+EGFR及其他靶向治疗

约50%的亚洲NSCLC患者携带*EGFR*基因突变。临床前研究显示^[30]^，EGFR基因活化突变可通过p-ERK1/2/p-c-Jun通路上调PD-L1在肿瘤细胞中的表达，从而诱导T细胞的凋亡。相反地，EGFR-酪氨酸激酶抑制剂（tyrosine kinase inhibitor, TKI）可解除EGFR-PD-1/PD-L1轴对T细胞的抑制效应，并增加IFN-γ的表达。EGFR-TKI耐药细胞系中PD-L1的表达水平显著较高，而PD-1抑制剂可降低EGFR-TKI耐药细胞系的生存能力^[30]^。PD-1抑制剂与EGFR抗体联合用药可能通过增强CD8^+^ T细胞的活性、增加DC的招募和聚集，从而提高免疫系统对肿瘤细胞的杀伤能力。因此，PD-1/PD-L1抑制剂与EGFR-TKI联合治疗NSCLC的研究正在逐步开展，如CheckMate 012（nivolumab+厄洛替尼）、KEYNOTE-021（pembrolizumab+厄洛替尼/吉非替尼）和NCT02088112（durvalumab+吉非替尼）。NCT02088112研究初步结果显示^[31]^，未经TKI治疗的EGFR敏感突变型NSCLC患者（*n*=19）接受durvalumab+吉非替尼联合治疗后ORR达77.8%-80.0%，提示PD-1抑制剂与EKGR-TKI的联合方案值得深入探索。但部分IO与靶向治疗联合方案的不良反应值得关注，CTLA-4抑制剂与靶向治疗的联合研究曾因剂量限制性毒性暂停招募^[32]^，*EGFR*突变阳性患者同时接受EGFR-TKI和PD-1抑制剂治疗时发生间质性肺炎的风险也显著升高（与单药治疗时相比OR=4.31，*P* < 0.01）^[33]^，因此，如何选择合适的治疗人群、是否能通过调整用药顺序或剂量克服不良反应，将成为今后该领域研究的挑战之一。

Swanson等^[34]^证实肺间质抗原提呈细胞生成的IDO在体内外均可对T细胞的免疫反应产生抑制效应。在多种肿瘤中，*IDO1*（编码IDO酶的基因）的过表达可使肿瘤逃避免疫监控和杀伤，并与患者生存预后较差相关。而对IDO1的抑制可减缓肿瘤生长、增强抗肿瘤免疫效应，可能与IO产生协同作用^[35]^。因此，IO与IDO1抑制剂的联合研究也已陆续开展，如KEYNOTE-654研究（pembrolizumab+ IDO抑制剂）^[36]^等。

### 3.5 IO+放疗

传统的分割放射治疗可与PD-1抑制剂联合，刺激CD8^+^ T细胞介导的肿瘤清除^[37]^。新兴的放疗技术立体定向放疗（stereotactic body radiation therapy, SBRT）亦可通过诱导主要组织相容性复合体、炎症因子、免疫调节因子和细胞粘附分子的表达，增强免疫系统的抗肿瘤效应。一项黑色素瘤与肾癌的动物模型研究^[38]^发现，PD-1抑制剂联合SBRT可使肿瘤原发灶消失，并产生“远隔效应”，使转移灶的肿瘤体积缩小66%。因此，PD-1/PD-L1抑制剂与放疗联合的协同效应也是值得期待的方向之一。Pembrolizumab+SBRT的后线联合研究PEMBRO-RT（*n*=72）^[39]^显示，pembrolizumab+SBRT较pembrolizumab单药治疗可显著延长晚期NSCLC患者的中位PFS（6.4个月*vs* 1.8个月；HR=0.55；*P*=0.04），提高患者ORR（41% *vs* 19%），但并不增加≥3级AE发生风险（17% *vs* 22%）。这一结果或许为未来IO+放疗一线研究的开展提供方向。ETOP NICOLAS Ⅱ期研究（*n*=49）^[40]^则在不可切除的局部晚期NSCLC患者中评估了nivolumab+同步放化疗一线方案的安全性，中位随访6.6个月的初步结果显示nivolumab+同步放化疗安全性良好，患者最常见的AE为疲劳和贫血，放疗后的3个月随访期内无≥3级肺炎事件发生。下一步预计将评估患者的1年PFS率作为有效性指标。目前在NSCLC中开展的IO+放疗联合研究还包括nivolumab+放疗（NCT02696993、NCT02831933）、pembrolizumab+放疗（NCT02587455、NCT02621398、NCT02303990、NCT02608385）和atezolizumab+放疗（NCT02400814）等^[41]^。

## 4 挑战与期望

IO使晚期NSCLC患者在传统治疗手段（如化疗及靶向治疗）的基础上进一步改善了治疗反应与生存预后，并避免了类似靶向治疗常见的耐药风险，但PD-1单药的人群选择限制使得绝大部分NSCLC患者都无法从IO一线单药治疗中获益。以PD-1/PD-L1抑制剂为基础的联合治疗可能产生协同作用，从而对更多NSCLC患者产生抗肿瘤效应，目前，有关PD-1/PD-L1抑制剂与其他检查点分子抑制剂、化疗、抗血管生成药物及其他靶向治疗等手段的联合治疗正成为NSCLC未来研究的热点方向。

多种联合用药方案的研究结果令人鼓舞，但亦有许多问题值得思考，包括：如何针对不同的联合方案筛选适宜人群、寻找可预测某一联合治疗方案疗效的精准生物标志物、探索联合用药的时机与顺序（同时或序贯用药）、联合方案的剂量选择与不良反应的标准处理流程、免疫治疗中特有的“假性进展”对疗效评估的误判影响等。

可以预见，未来NSCLC的一线免疫治疗将出现多种选择，包括IO单药、IO联合IO、IO联合化疗及IO联合靶向治疗等。如何根据合适的标志物（如PD-L1表达状态、肿瘤突变负荷等）排兵布阵，为合适的患者选择最为合适的治疗，从而使患者获益最大化，已成为目前最值得思考的问题。此外，除了现有的联合治疗方案，还有很多与肿瘤微环境免疫抑制机制相关的联合方向值得探索。因此，IO联合治疗在NSCLC中的临床应用与探索之路仍任重道远。

## References

[1] Pardoll DM (2012). The blockade of immune checkpoints in cancer immunotherapy. Nat Rev Cancer.

[2] Iwai Y, Ishida M, Tanaka Y (2002). Involvement of PD-L1 on tumor cells in the escape from host immune system and tumor immunotherapy by PD-L1 blockade. Proc Natl Acad Sci U S A.

[3] Buchbinder EI, Desai A (2016). CTLA-4 and PD-1 pathways: Similarities, differences, and implications of their inhibition. Am J Clin Oncol.

[4] Ji M, Liu Y, Li Q (2015). PD-1/PD-L1 pathway in non-small-cell lung cancer and its relation with *EGFR* mutation. J Transl Med.

[5] Brahmer J, Horn L, Jackman D (2017). Five-year follow-up from the CA209-003 study of nivolumab in previously treated advanced non-small cell lung cancer: Clinical characteristics of long-term survivors. AACR Annual Meeting.

[6] Malhotra J, Jabbour SK, Aisner J (2017). Current state of immunotherapy for non-small cell lung cancer. Transl Lung Cancer Res.

[7] Ettinger DS, Wood DE, Aisner DL (2017). Non-small cell lung cancer, Version 5.2017, NCCN Clinical Practice Guidelines in Oncology. J Natl Compr Canc Netw.

[8] Remon J, Besse B, Soria JC (2017). Successes and failures: what did we learn from recent first-line treatment immunotherapy trials in non-small cell lung cancer?. BMC Med.

[9] Santabarbara G, Maione P, Rossi A (2016). The role of pembrolizumab in the treatment of advanced non-small cell lung cancer. Ann Transl Med.

[10] Curran MA, Montalvo W, Yagita H (2010). PD-1 and CTLA-4 combination blockade expands infiltrating T cells and reduces regulatory T and myeloid cells within B16 melanoma tumors. Proc Natl Acad Sci U S A.

[11] Hellmann MD, Rizvi NA, Goldman JW (2017). Nivolumab plus ipilimumab as first-line treatment for advanced non-small-cell lung cancer (CheckMate 012): results of an open-label, phase 1, multicohort study. Lancet Oncol.

[12] Goldman JW, Antonia SJ, Gettinger SN (2017). Nivolumab (N) plus ipilimumab (I) as first-line (1L) treatment for advanced (adv) NSCLC: 2-yr OS and long-term outcomes from CheckMate 012. J Clin Oncol.

[13] Goldman JW, Antonia SJ, Gettinger SN (2017). Nivolumab plus ipilimumab as first-line treatment for advanced NSCLC: 2-year overall survival and long-term outcomes from CheckMate 012. 2017 American Society Clinical Oncology Annual Meeting.

[b14] 14Hellmann M D, Ciuleanu TE, Pluzansk i A, et al. Nivolumab plus ipilimumab in lung cancer with a high tumor mutational burden. N Engl J Med, 2018, 378: 2093-2104. doi: 10.1056/NEJMoa1801946

[15] Antonia S, Goldberg SB, Balmanoukian A (2016). Safety and antitumour activity of durvalumab plus tremelimumab in non-small cell lung cancer: a multicentre, phase 1b study. Lancet Oncol.

[16] Emens LA, Middleton G (2015). The interplay of immunotherapy and chemotherapy: harnessing potential synergies. Cancer Immunol Res.

[17] Obeid M, Tesniere A, Ghiringhelli F (2007). Calreticulin exposure dictates the immunogenicity of cancer cell death. Nat Med.

[18] Gentzler RD, Langer CJ, Borghaei H (2018). 24-month overall survival from KEYNOTE-021 cohort G: Pemetrexed-carboplatin plus pembrolizumab as first-line therapy for advanced nonsquamous NSCLC. J Clin Oncol.

[19] Langer CJ, Gadgeel SM, Borghaei H (2016). Carboplatin and pemetrexed with or without pembrolizumab for advanced, non-squamous non-small-cell lung cancer: a randomised, phase 2 cohort of the open-label KEYNOTE-021 study. Lancet Oncol.

[20] Gandhi L, Rodriguez-Abreu D, Gadgeel S (2018). Pembrolizumab plus chemotherapy in metastatic non-small-cell lung cancer. N Engl J Med.

[21] Paz-Ares LG, Alexander L, Tafreshi A (2018). Phase 3 study of carboplatin-paclitaxel/nab-paclitaxel (Chemo) with or without pembrolizumab (Pembro) for patients (Pts) with metastatic squamous (Sq) non-small cell lung cancer (NSCLC). J Clin Oncol.

[22] Rizvi NA, Hellmann MD, Brahmer JR (2016). Nivolumab in combination with platinum-based doublet chemotherapy for first-line treatment of advanced non-small-cell lung cancer. J Clin Oncol.

[23] Juergens R, Hellmann M, Brahmer J (2017). OA 17.03 first-line Nivolumab plus platinum-based doublet chemotherapy for advanced NSCLC: CheckMate 012 3-year update. J Thorac Oncol.

[24] Paz-Ares L (2017). CheckMate 227: A randomized, open-label phase 3 trial of nivolumab, nivolumab plus ipilimumab, or nivolumab plus chemotherapy versus chemotherapy. Ann Oncol.

[25] Liu SV, Camidge DR, Gettinger SN (2017). Atezolizumab (atezo) plus platinum-based chemotherapy (chemo) in non-small cell lung cancer (NSCLC): Update from a phase Ib study. J Clin Oncol.

[26] Jotte RM, Cappuzzo F, Vynnychenko I (2018). IMpower131: Primary PFS and safety analysis of a randomized phase Ⅲ study of atezolizumab +carboplatin+paclitaxel or nab-paclitaxel vs carboplatin+nab-paclitaxel as 1L therapy in advanced squamous NSCLC. J Clin Oncol.

[27] Ott PA, Hodi FS, Buchbinder EI (2015). Inhibition of immune checkpoints and vascular endothelial growth factor as combination therapy for metastatic melanoma: An overview of rationale, preclinical evidence, and initial clinical data. Front Oncol.

[28] Reck M, Socinski MA, Cappuzzo F (2017). Primary PFS and safety analyses of a randomized phase Ⅲ study of carboplatin+paclitaxel +/- bevacizumab, with or without atezolizumab in 1L non-squamous metastatic NSCLC (IMpower150). Ann Oncol.

[29] Kowanetz M, Socinski MA, Zou W (2018). IMpower150: Efficacy of atezolizumab plus bevacizumab and chemotherapy in 1L metastatic nonsquamous NSCLC across key subgroups. AACR Annual Meeting Proceedings.

[30] Chen N, Fang W, Zhan J (2015). Upregulation of PD-L1 by EGFR activation mediates the immune escape in EGFR-driven NSCLC: Implication for optional immune targeted therapy for NSCLC patients with *EGFR* mutation. J Thorac Oncol.

[31] Gibbons DL, Chow LQ, Kim DW (2016). 57O efficacy, safety and tolerability of MEDI4736 (durvalumab[D]), a human IgG1 anti-programmed cell death-ligand-1 (PD-L1) antibody, combined with gefitinib (G): A phase Ⅰ expansion in TKI-naive patients (pts) with EGFR mutant NSCLC. J Thorac Oncol.

[32] Morrissey KM, Yuraszeck TM, Li CC (2016). Immunotherapy and novel combinations in oncology: Current landscape, challenges, and opportunities. Clin Transl Sci.

[33] Oshima Y, Tanimoto T, Yuji K (2018). EGFR-TKI-associated interstitial pneumonitis in nivolumab-treated patients with non-small cell lung cancer. JAMA Oncol.

[34] Swanson KA, Zheng Y, Heidler KM (2004). CDllc^+^ cells modulate pulmonary immune responses by production of indoleamine 2, 3-dioxygenase. Am J Respir Cell Mol Biol.

[35] Spranger S, Koblish HK, Horton B (2014). Mechanism of tumor rejection with doublets of CTLA-4, PD-1/PD-L1, or IDO blockade involves restored IL-2 production and proliferation of CD8(+) T cells directly within the tumor microenvironment. J Immunother Cancer.

[36] Awad MM, Munteanu M, Zhao YF (2018). ECHO-305/KEYNOTE-654: A phase 3, randomized, double-blind study of first-line epacadostat plus pembrolizumab *vs* pembrolizumab plus placebo for metastatic non-small cell lung cancer (mNSCLC) with high PD-L1 levels. J Clin Oncol.

[37] Martinov T, Fife BT (2016). Fractionated radiotherapy combined with PD-1 pathway blockade promotes CD8 T cell-mediated tumor clearance for the treatment of advanced malignancies. Ann Transl Med.

[38] Park SS, Dong H, Liu X (2015). PD-1 restrains radiotherapy-induced abscopal effect. Cancer Immunol Res.

[39] Theelen W, Peulen H, Vries J (2018). Randomized phase Ⅱ study of pembrolizumab after stereotactic body radiotherapy (SBRT) versus pembrolizumab alone in patients with advanced non-small cell lung cancer: The PEMBRO-RT study. J Clin Oncol.

[40] Peters S, Ruysscher DD, Dafni U (2018). Safety evaluation of nivolumab added concurrently to radiotherapy in a standard first line chemo-RT regimen in unresectable locally advanced NSCLC: The ETOP NICOLAS phase Ⅱ trial. J Clin Oncol.

[41] Lazzari C, Karachaliou N, Bulotta A (2018). Combination of immunotherapy with chemotherapy and radiotherapy in lung cancer: is this the beginning of the end for cancer?. Ther Adv Med Oncol.

